# In-hospital cardiac arrest in piedmont (ITALY): epidemiology and outcomes

**DOI:** 10.1186/2197-425X-3-S1-A197

**Published:** 2015-10-01

**Authors:** A Mira, G Radeschi, G Berta, F Rubulotta

**Affiliations:** Monitoring Centre for In-Hospital Emergency of the Piedmont Region - S. Luigi Gonzaga Hospital, ICU, Orbassano, Italy; Monitoring Centre for In-Hospital Emergency of the Piedmont Region - S. Luigi Gonzaga Hospital, Orbassano, Italy; Imperial College London, London, United Kingdom

## Introduction

Hospitals situated in the Piedmont Region (Italy) have systematically collected data on in-hospital cardiac arrest (IHCA) since 2012. This activity has been established as part of the Regional Guidelines for the continuous quality improvement of the in-hospital emergency. Those guidelines and improvement strategies are coordinated by the Scientific Committee of the Regional Board and transferred to the Monitoring Center (CdM) that takes care of the data quality control, and the preparation of an annual report.

## Objectives

The aim of this study is to describe the epidemiology, nature, incidence and clinical outcomes of IHCA over twelve consecutive months (January 1^st^ to December 31^st^, 2013) in Piedmont Region (35 Hospitals, 618 consecutive patients). Our database analysis could be valuable for comparison/benchmarking with national and international data/standards ^(1)^.

## Methods

Systematic data collection was performed using the Italian Cardiac Arrest Register (RIAC). This is an electronic online database created by the Italian Resuscitation Council (IRC). Within the database, the 35 Piedmont hospitals form a cluster. The records were collected using the Utstein standard. Ethics approval was obtained by the local ethics review board and all patient´ information were collected anonymously and analyzed by the CdM.

## Results

The CdM reviewed over 12 months a total of 618 patients in 25 centers. The patients´ characteristics are shown in Table 1 and the outcomes in Table 2. The incidence of IHCA was 1.7/1000 admissions/year. The presenting rhythm was shockable in 23,1% and ROSC was obtained in 228 cases (41,1%). The survivors at hospital discharge were 79 (14,2% of the CPR started), 73 of those (92,4%) with good neurological outcomes (CPC 1-2). Follow-up at 6 months showed 60 patients (10,8%) are still alive.

## Conclusions

35 hospitals collaborated in Piedmont collecting data regarding IHCA. The incidence of IHCA is comparable with one of the most recent international studies^(1)^. Major limitation of this study is the fact that we recorded only the events involving the MET. Therefore, we cannot exclude other events occurred without involving the medical emergency team. The percentage of shockable rhythms was slightly lower than the average reported in the literature. We believe that epidemiological data and outcome data recorded for IHCA in Piedmont could be compared and benchmark with other centers at a national and international level. Therefore, we are confident that this study will help improving the treatment of cardiac arrest and the organization of the emergency systems.
